# Comparison of Dry Needling versus Orthopedic Manual Therapy in Patients with Myofascial Chronic Neck Pain: A Single-Blind, Randomized Pilot Study

**DOI:** 10.1155/2015/327307

**Published:** 2015-11-10

**Authors:** Irene Campa-Moran, Etelvina Rey-Gudin, Josué Fernández-Carnero, Alba Paris-Alemany, Alfonso Gil-Martinez, Sergio Lerma Lara, Almudena Prieto-Baquero, José Luis Alonso-Perez, Roy La Touche

**Affiliations:** ^1^Faculty of Health Science, Department of Physiotherapy, The Center for Advanced Studies University La Salle, Faculty of Health Science, The Autonomous University of Madrid, Aravaca, Madrid, Spain; ^2^Department of Physical Therapy, Occupational Therapy, Rehabilitation and Physical Medicine, King Juan Carlos University, Alcorcón, Madrid, Spain; ^3^Hospital La Paz Institute for Health Research, IdiPAZ, Madrid, Spain; ^4^Motion in Brains Research Group, The Center for Advanced Studies University La Salle, The Autonomous University of Madrid, Spain; ^5^Institute of Neuroscience and Craniofacial Pain (INDCRAN), Madrid, Spain; ^6^Movement Analysis Laboratory, University Hospital Niño Jesus, Madrid, Spain; ^7^Department of Physiotherapy, European University of Madrid, Villaviciosa de Odón, Madrid, Spain

## Abstract

*Objective*. The aim of this study was to compare the efficacy of three interventions for the treatment of myofascial chronic neck pain. *Methods*. Thirty-six patients were randomly assigned to one of three intervention groups: orthopedic manual therapy (OMT), dry needling and stretching (DN-S), and soft tissue techniques (STT). All groups received two treatment sessions with a 48 h time interval. Outcome measures included neck pain intensity measured using a visual analogue scale, cervical range of motion (ROM), pressure pain threshold for measuring mechanical hyperalgesia, and two self-reported questionnaires (neck disability index and pain catastrophizing scale). *Results*. The ANOVA revealed significant differences for the group × time interaction for neck disability, neck pain intensity, and pain catastrophizing. The DN-S and OMT groups reduced neck disability. Only the OMT group showed decreases in mechanical hyperalgesia and pain catastrophizing. The cervical ROM increased in OMT (i.e., flexion, side-bending, and rotation) and DN-S (i.e., side-bending and rotation) groups. *Conclusions*. The three interventions are all effective in reducing pain intensity. Reduction in mechanical hyperalgesia and pain catastrophizing was only observed in the OMT group. Cervical ROM improved in the DN-S and OMT groups and also neck disability being only clinically relevant for OMT group.

## 1. Introduction

Neck pain is one of the most frequent pain conditions; the prevalence of neck pain in the general population has been estimated between 10% and 15%, being more common in females than in males [1]. In a recent study, the prevalence of neck pain in the adult Spanish population has been estimated at 19.5% [2].

There have been many studies to determine the causes of neck pain, but there remains a lack of knowledge about the etiology of this condition. Myofascial pain (MP) is a common variety of such pain, usually caused by myofascial trigger points (MTrPs) [3]. The MTrPs in the neck muscles have been associated with a possible source of referred facial and cranial pain [3].

Myofascial trigger points (MTrPs) in the muscles of the neck and shoulder often coexist with neck pain conditions and can contribute to the symptoms [4]. Therefore, there is increasing evidence that MTrPs often play a role in the symptoms of patients with neck pain [5]. The muscle most often affected with the presence of MTrPs in the neck region is the trapezius muscle [6, 7], specifically the upper fibers, and this is the most hyperalgesic muscle of the neck and shoulder [8].

Active MTrPs are defined as hyperirritable spots within a taut band in skeletal muscles that are painful on compression and when stimulated (by digital compression or dry needling) can evoke a characteristic pattern of referred pain and related autonomic phenomena [3]. These findings are supported by other outcomes such as altered EMG activity, which has also been associated with chronic neck pain [9, 10]. In other recent studies, an altered motor control strategy of the trapezius muscle during isometric shoulder exercise has been reported [11].

Other major locations responsible for neck pain in patients are the articular zygapophysial joints (facet joints) [12]. Some studies have determined the prevalence of painful zygapophysial joints in patients with neck pain by blocking the medial branch of the spinal dorsal horn, with very positive results for the reduction of pain [12].

The majority of patients with chronic neck pain are treated in primary care by either medical intervention or physiotherapy [13]. Manual therapy, such as manipulation and mobilization, is frequently used for the treatment for neck pain and has great support in Cochrane reviews [14]. The preferred techniques for the treatment of articular origin pain are the manipulation or mobilization of the affected segments; the handling achieves a more immediate effect in reducing the pain, whereas the mobilization effect acts in the medium-to-long term [15, 16]. It has been shown that the mobilization has an effect on pain perception in patients with neck pain [17, 18]. Neurophysiologic effects have been demonstrated after mobilization techniques have been applied over the cervical spine [18]. However, a recent systematic review [14] reported that there is a lack of evidence to allow definitive conclusions about which groups of patients will benefit the most from what type of manual therapy technique (manipulation or mobilization) applied.

Many forms of treatment, such as ischemic compression [19, 20] and dry needling [21–24], have shown positive effects of reduction of pain intensity when they are applied at the MTrPs in the trapezius muscle of patients with neck pain. Furthermore, in a systematic review, it was concluded that the effect of injection therapies is likely to be due to the physical prick of the needle rather than to the type of substance injected [25].

Experts recommend that a multimodal approach must be investigated in patients with neck pain [26], because the clinical patterns of patients often do not respond to a single intervention, but they do with a multimodal approach. This is because, in real cases, patients with neck pain may present several disorders, and the symptoms of the patients therefore do not automatically respond to a single treatment.

In our investigation, a randomized clinical trial was designed to respond to three objectives: (1) to compare the efficacy of orthopedic manual therapy (OMT), dry needling, stretching (DN-S), and soft tissue techniques (STT) for the treatment of MTrP in patients with myofascial chronic neck pain, (2) to compare the treatment effects over disability and catastrophizing in patients with neck pain, and finally (3) to evaluate the likely neurophysiological effects produced by these techniques in the neck.

## 2. Materials and Methods

### 2.1. Research Design

A randomized, single-blind study design was used. An independent “blind” assessor made the measurements and registered the data. Patients were randomly allocated to one of three different intervention groups (DN-S group, STT group, and OMT group) performed by a computer-generated random-sequence table created before the start of the study with GraphPad software (GraphPad Software, Inc., CA 92037, USA).

### 2.2. Participants

Participants were recruited from the Public Valleagudo Primary Health Care Center in Coslada, Madrid, Spain. Patients with cervical pain of muscular origin were referred and screened for possible eligibility criteria. In this research we defined cervical pain as mechanical pain in the cervical muscles, which can be provoked both by maintained postures and by movements. Patients were selected if they met all of the following criteria: (a) bilateral pain involving the upper trapezius and elevator muscle of the scapula; (b) a duration of pain of at least 3 months; (c) a pain intensity corresponding to at least 20 mm on a 100 mm visual analogue scale (VAS); (d) neck pain with symptoms provoked by either neck postures or neck movement; (e) pain localized at least in the cervical and occipital regions but not in the orofacial region; (f) neck disability index (NDI) [27, 28] greater than or equal to 15 points; (g) restricted cervical range of movements (flexion, extension, rotation, and side-bending); (h) presence of bilateral MTrPs in upper trapezius and levator scapulae muscles. MTrPs were diagnosed according to the following criteria [29]: (1) presence of a palpable taut band in skeletal muscle, (2) presence of a hypersensitive tender spot within this taut band, and (3) reproduction of referred pain in response to MTrP compression. In order to meet the criteria to participate in the study, patients had to pass an initial physical examination performed by a single investigator to rule out the presence of nerve root compression. (i) Patients were between 18 and 75 years of age.

Patients were excluded if they presented any signs, symptoms, or history of the following diseases: (a) orofacial pain and temporomandibular disorders according to the Research Diagnostic Criteria of Temporomandibular Disorders (RDC/TMD); (b) a history of traumatic injuries (e.g., contusion, fracture, and whiplash injury); (c) systemic diseases such as fibromyalgia, systemic erythematous lupus, and psoriatic arthritis; (d) neurologic disorders (e.g., trigeminal neuralgia or occipital neuralgia); (e) concomitant medical diagnosis of any primary headache (tension type or migraine); (f) unilateral neck pain; (g) cervical spine surgery; (h) clinical diagnosis of cervical radiculopathy or myelopathy; (i) needle phobia; (j) history of previous physical therapy intervention for the cervical region.

Each participant received a thorough explanation about the content and purpose of the treatment before signing an informed consent form relative to the procedures. All of the procedures used in this study were planned under the ethical norms of the Helsinki Declaration and were approved by the local ethics committee.

### 2.3. Demographic and Clinical Data

Each of the eligible patients had to complete a basic questionnaire to determine if they met the criteria for inclusion or exclusion. This questionnaire included demographic data (gender, age, height, and weight), a body chart where patients had to mark the location of their pain, and several questions about their characteristics of their pain; for example, when did it start? How does it worsen or alleviate? What kind of pain is it?

### 2.4. Self-Report Questionnaires

The following questionnaires have been used to asses psychological and disability variables: the Spanish version of the Pain Catastrophizing Scale (PCS) [30, 31] was used to measure the catastrophic level associated with pain and the Spanish version of the NDI [27, 28] to measure perceived neck disability in order to quantify the psychophysical state of the patients. All patients included in the research had to complete all these questionnaires before the first intervention and a week after the last intervention (follow-up period).

#### 2.4.1. Pain Catastrophizing

The PCS instructions ask participants to reflect on past painful experiences and to indicate the degree to which they experienced each of 13 thoughts or feelings (items) when experiencing pain. Items are scored on a 5-point scale with the end points “0” (not at all) and “4” (all the time). The PCS yields a total score ranging from 0 to 52. Three subscale scores can be obtained assessing rumination, magnification, and helplessness. The Spanish version of the PCS has been used in this research. It also showed appropriate internal consistency (Cronbach alpha = 0.79) and test-retest reliability (intraclass correlation coefficient = 0.84) [31].

#### 2.4.2. Neck Disability Index (NDI)

It consists of 10 items assessing different functional activities and uses a 6-point Likert scale ranging from 0 (no disability) to 5 (complete disability). The overall score (out of 100) is obtained by adding the score for each item and multiplying by 2. A higher score indicates greater pain and disability. The validated Spanish version was used in this study [27]. The minimum detectable change is 5 points out of 50, and it is recommended that 7 points is the minimum clinically important difference [32].

### 2.5. Outcome Measures

Outcome measures included neck pain intensity measured with VAS, the cervical range motion (ROM), and the pressure pain threshold (PPT) for measuring the mechanical hyperalgesia. Measurements were performed before the first treatment session (baseline), after the first session (post 1), after the second session (post 2), and one week after the last measurement (follow-up period).

#### 2.5.1. Visual Analogue Scale (VAS)

A VAS was used to measure pain intensity before and after each treatment. The VAS comprises a 100 mm horizontal line from 0 mm representing “no pain” to 100 mm representing “pain as bad as you can imagine”. The patient placed a mark on the line at the point that they felt represented their intensity at the time, which was quantified by the assessor in mm. This scale has proven its reliability and validity for the measurement of pain intensity [33].

#### 2.5.2. Pressure Pain Threshold (PPT)

PPT is defined as the minimum amount of pressure needed to provoke a pain sensation [34]. We used a digital algometer (Model FDX 10, Wagner Instruments, Greenwich, CT, USA) that consists of a rubber head (1 cm^2^) attached to a pressure gauge which measures in kg, with thresholds expressed in kg/cm^2^. The protocol was a sequence of three measurements, with an interval of 30 seconds between each. The average of the three outcomes was calculated in order to obtain only one value for all the measured points in each of the assessments. This algometric method has a high reliability (ICC = 0.91, IC of 95%: 0.82–0.97) for PPT measurement. PPT were assessed at one point in the C5 zygapophysial joint and upper trapezius, all of them bilaterally. The device was applied perpendicular to the skin. The patients were asked to raise their hand at the moment the pressure started to change to a pain sensation, at which point the assessor stopped applying pressure. This sequence was performed three times. Anatomic references for the algometric measurements were C5 zygapophysial joint: 2 cm lateral to the spinous process of C6; trapezius muscle: 2.5 cm above the superior medial angle of the scapula.

#### 2.5.3. Cervical Range of Motion (CROM)

Cervical ROM was measured with a cervical goniometer called a CROM. This device has three inclinometers, one at each plane of movement. A plastic glasses-like support houses two of the inclinometers which allows the measurement of flexion, extension, and side-bending of the neck. Adding another part of the device with the third inclinometer and the magnets around the neck allows rotations to be measured. The subjects were instructed to sit upright, relax their shoulders, and rest their hands on their thighs, with hips and knees flexed at 90°. Different verbal commands were given to the subjects to perform the neck movements properly. For flexion, “bring your chin downwards and then tilt your head forwards as far as possible”; for extension, “bring your chin up and then tilt your head backward as far as possible"; for side-bending, “bring your ear toward your shoulder as far as possible, maintaining your face to the front and not moving your shoulder”; for rotations, “turn your head to the side without moving your shoulders”. All movements had to be done without pain. In some of the subjects the movement had to be hand-guided to achieve a proper movement. This procedure has demonstrated its reliability in patients with neck pain [35].

### 2.6. Procedure

Once the subjects went through the final assessment and were included in the research, a randomization into three groups was performed. The DN-S group received dry needling and stretching; the STT group received soft tissue techniques treatment focused on the muscle; the OMT group received an OMT focused in joints and nerves. All groups received two treatment sessions, with a 48 h interval between them.

#### 2.6.1. DN-S Group

DN-S group received two treatments of bilateral dry needling on levator scapulae and upper trapezius muscles and a passive stretching technique. We chose those muscles because they are more affected in patients with neck pain [36]. The needling technique applied was performed according to the method of Hong et al. The needles used were 0.26 × 25 mm. The technique began with palpation of the active MTrP localizing the more sensitive taught band of the muscle. The needle was inserted in the direction of the taught band and perpendicular to the skin and was directed to the muscle MTrP until a first local twitch response was provoked. Then, the needle was inserted and withdrawn; the local twitch response was perceived by the therapist as a transient and involuntary contraction of the taut band. The needle insertions were repeated to achieve at least three local twitch responses. Then, the needle was withdrawn. The needling procedure at each MTrP lasted about 2 minutes. Once the needle was withdrawn, firm compression was exerted on the insertion site for 40 seconds to avoid excessive bleeding.

Following the needling procedure, a passive stretching to the levator scapulae and trapezius muscles was applied bilaterally for 20 seconds to each muscle.

#### 2.6.2. STT Group

These patients received a bilateral OMT treatment based on the ischemic compression technique over both the levator scapulae and upper trapezius muscles, but also a dynamic soft tissue mobilization (DSTM) was applied on the upper trapezius for four minutes. For the ischemic compression technique, the physiotherapist (PT) applied gradually increasing pressure to the MTrP until the patient felt the sensation of pressure changed into pain; at that time the pressure was maintained until the discomfort eased, at which moment the pressure was increased until discomfort was again perceived by the patient. This process was repeated for 90 s while the patient was lying prone. This technique has been used in previous studies [19, 20].

The DSTM are a group of techniques used to treat the muscle, a direct stimulus over a specific region of the muscle (pressure, gliding pressure, etc.) added to stretching of the muscle or a mobilization of the closest joint or both together.

For group 2, a DSTM over the trapezius muscle was used. The patient was in lateral decubitus; the PT positioned one hand over the acromion and the other hand at the distal part of the upper trapezius. The technique consisted of performing a circular movement of the scapular belt while a slow gliding pressure was applied over the trapezius muscle in the direction to the occipital bone while the muscle was in a relaxed position. When the shoulder was depressed and therefore the muscle was stretched, nothing was performed over the muscle but the stretching itself. The technique was applied bilaterally for two minutes.

#### 2.6.3. OMT Group

OMT group received an OMT protocol with a neural/joint approach, with three techniques: (1) anterior-posterior upper cervical mobilization (APUCM) with wedge (four min); (2) the cervical lateral glide mobilization technique at C4 and C5 (two min each side); and (3) neural thoracic mobilization with wedge (four min):

1. APUCM: with the patient lying supine with a neutral position of the cervical spine, the wedge was positioned under the C2 spinous process. The PT held the occipital region of the patient with both hands to stabilize and maintain the position of the upper cervical structures, while with the anterior part of his shoulder applying a posteriorly directed force on the frontal region of the patient (anterior to posterior force). The mobilization was applied at a slow rate of one oscillation per two seconds (0.5 Hz) controlled with a digital metronome MA-30 (Korg Inc., Japan). The total time of mobilization was four minutes, applied for two intervals of two minutes each, with 30 seconds rest in between. This technique has been used in previous studies [37].
2. Cervical lateral glide mobilization technique: with the patient in a supine position, the PT cradled the head and neck of the patient and, including the levels to be treated (C4-C5), performed a lateral translatory movement while minimizing gross cervical side flexion or rotation [38, 39], spending two min at each point and side and a total of eight min. This technique has been used in previous studies [38–40].
3. Neural thoracic mobilization: patient was lying supine, with both knees in flexion and one leg crossed over the other, maintaining the knees together. A wedge is placed under the patient's back, with the upper side at T4-T5 level. The PT holds the head with the forearm in a craniocervical flexion and submaximal cervical flexion; the hand is placed under the spine at the mobilization level, to ensure the vertebrae are mobilizing. A towel is placed over the sternum of the patient and the other hand of the PT is placed over the towel to exert an anterior-posterior pressure. This is a dynamic technique; the patient is asked to extend the crossed leg without losing the knee-knee contact, and when the patient again flexed the knee, the PT applied the pressure over the sternum.

### 2.7. Statistical Analysis

Data were analyzed using the SPSS software package, version 19 (SPSS, Chicago, IL). Normal distribution was confirmed with the Shapiro-Wilk test (*P* > 0.05) for NDI, PCS, and VAS variables, so parametric statistics were used. Means and standard deviations were calculated for each variable. The baseline and demographic data at pretreatment were compared among groups using a one-way analysis of variance (ANOVA). A 2-way repeated measures ANOVA was performed to evaluate the effect in all the variables, with intervention (DN-S group 1, STT group 2, and OMT group 3) and time (before treatment, immediately after the first session, after the second session, and one week after treatment) as factors. Tests of within-patients* post hoc* simple effects (i.e., changes in time for all variables for each group separately) were performed with Bonferroni corrections. Partial eta-squared (*η*_*p*_^2^) was calculated as a measure of effect size (strength of association) for each main effect and interaction in the ANOVAs, with 0.01–0.059 representing a small effect, 0.06–0.139 a medium effect, and > 0.14 a large effect [41].

The PPTs and CROM variables did not meet the normal distribution test, so nonparametric statistics were used. Descriptive statistics were used to summarize the data, including means and SDs, medians, and interquartile ranges (IQR) for continuous data. The Kruskal-Wallis test was used for the analysis and was used to compare data among the three groups to baseline data. The Friedman test was used to analyze the change from the intragroup results, and the Wilcoxon signed rank test was used for* post hoc* intragroup comparisons. In all statistical comparisons, *P* < 0.05 was used as the criterion for statistical significance.

## 3. Results

**Figure 1:**
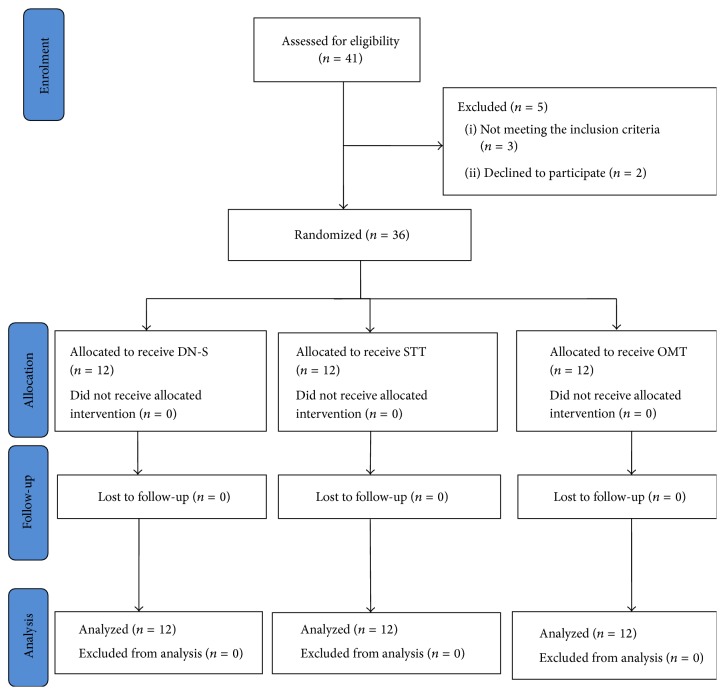
Flow fiagram.

Thirty-six patients (7 males and 29 females) with chronic mechanical neck pain, aged 18 to 73 years (mean 49.5 ± 13.05 years; mean height 160 ± 7 cm; mean weight 66 ± 13 kg), were included in this study and assigned to one of three groups (Figure 1). No significant differences were found between groups for age (*F* = 1.19, *P* = 0.31), height (*F* = 0.6, *P* = 0.55), weight (*F* = 2.72, *P* = 0.08), and NDI (*F* = 0.19, *P* = 0.82), and pain duration (*F* = 2.1, *P* = 0.13). However, significant differences between groups appeared for pain intensity (*F* = 3.38, *P* = 0.04). Demographic and clinical data for each group are detailed in Table 1. There were no subject drop-outs during the different phases of the study, and no adverse effects were registered after the application of the three treatments. None of the subjects started drug therapy during the lifetime of the study.

### 3.1. Self-Report Psychological Questionnaires: Catastrophizing and Neck Disability Outcomes

The ANOVA revealed significant differences for time factor (*F* = 35.29, *P* < 0.000; *η*_*p*_^2^ = 0.51) and for group × time interaction (*F* = 3.84, *P* = 0.032; *η*_*p*_^2^ = 0.19) for neck disability. Statistical descriptive data and* post hoc* results are represented in Table 2. For catastrophizing, differences were found for time factor (*F* = 10.45, *P* = 0.003; *η*_*p*_^2^ = 0.24) and for group × time interaction (*F* = 3.44, *P* = 0.04; *η*_*p*_^2^ = 0.17).

The* post hoc* analysis showed significant differences in the comparison between pretreatment and follow-up period (*P* < 0.001) for neck disability for groups 2 and 3. Descriptive data and* post hoc* results are shown in Table 2.

The* post hoc* analysis showed significant differences in the comparison between pretreatment and follow-up period (*P* < 0.001) for pain catastrophizing for group 3. Descriptive data and* post hoc* results are shown in Table 2.

### 3.2. Visual Analogue Scale

The ANOVA revealed that significant differences are present for time factor (*F* = 25.8, *P* < 0.001; *η*_*p*_^2^ = 0.44) and group × time interaction (*F* = 5.0, *P* < 0.001; *η*_*p*_^2^ = 0.23) regarding pain intensity. The* post hoc* analysis found significant differences between pretreatment, post 1, post 2, and follow-up data (*P* < 0.05) for pain intensity in group 2 and group 3 (Table 3). However, regarding group 1, differences were found in the comparisons of pretreatment with the follow-up period (*P* = 0.001), post 1 with follow-up period (*P* < 0.001), and post 2 with follow-up period (*P* < 0.001).

### 3.3. Pressure Pain Thresholds in the Cervical Region

In the Kruskal-Wallis test, differences were found for PPT in C5-C6 in post 1, post 2, and follow-up at two weeks (*P* < 0.05) and for PPT in trapezius muscle in the baseline, post 1, and follow-up at two weeks (*P* < 0.05). The Wilcoxon test showed significant differences for PPT in C5-C6 and PPT over trapezius outcomes when the baseline data were compared with post 1, post 2, and follow-up at two weeks for group OMT, but not for the groups DN-S and STT. The Friedman test showed that, in the group OMT, the PPT over C5-C6 and trapezius variables were found to be statistically significant (*P* < 0.01), but not for groups DN-S and STT. Comparisons between groups 1 and 3 at follow-up and between groups 2 and 3 in post 1, post 2, and the follow-up period for PPT over C5-C6 and between groups 1 and 2 at follow-up and between groups 2 and 3 in post 1, post 2, and follow-up period for PPT over trapezius were examined using the Mann-Whitney *U* test. The results for PPT are presented in Table 4.

### 3.4. Active Cervical Range of Motion Assessment

In the Kruskal-Wallis test, differences were found for flexion and for extension in the follow-up at two weeks (*P* < 0.05). The Wilcoxon test showed significant differences for flexion outcomes when the baseline data were compared with follow-up at two weeks for group 1 and when the baseline data were compared with post 1, post 2, and follow-up at two weeks for group 3, but not for group 2 (*P* < 0.05). Significant differences were observed for side-bending outcomes when the baseline data were compared with post 2 and follow-up at 2 weeks for group 1 and when the baseline data were compared with post 2 for group 2, but not for group 3 (*P* < 0.05), and for rotation outcomes when the baseline data were compared with post 2 for group 1 and when the baseline data were compared with post 2 and with follow-up for group 3, but not for group 2 (*P* < 0.05). The Friedman test showed that, in group 3, the flexion was found to be statistically significant (*P* < 0.01), but not for groups 1 and 2, and, in groups 1 and 3, the side-bending and rotation were found to be statistically significant (*P* < 0.01), but not for group 2. In comparisons between groups 1 and 3 at post 2 and follow-up and between groups 2 and 3 in follow-up period for flexion, between groups 1 and 3 at post 2 and follow-up and between groups 2 and 3 in follow-up period for extension, and between groups 1 and 3 at post 2 for rotation, using the Mann-Whitney *U* test, the results for CROM are presented in Tables 5 and 6.

## 4. Discussion

In the present study all groups experienced a statistically significant improvement in neck pain intensity 1 week after intervention in patients with myofascial chronic neck pain. However, only the OMT and DN-S had an immediate effect reducing neck pain intensity after the first and second sessions. In addition, only the OMT group had a short term hypoalgesic effect for reducing the catastrophizing level and for increasing of CROM for flexion and extension, but not the DN-S and STT groups. The DN-S and OMT groups showed a short term effect reducing neck disability, but not the STT group. There were no differences in side-bending and rotation movements between groups, but the DN-S and OMT had an increase of range of motion higher than the STT group.

### 4.1. Neck Pain Intensity

The effects of pain reduction produced by the three groups were at their strongest two weeks after treatment (mean difference −20.5 mm, −15.9 mm, and −32.7 mm for DN-S, STT, and OMT, resp.) indicating that the changes are clinically relevant, since the magnitude of change of clinically important difference is established in 8.5 mm [42]. The pain reduction represents a change of 39.3% for the DN-S group, 68.3% for the STT group, and 89% for the OMT group. Changes of 30% or more are considered to be clinically meaningful improvements in spinal pain conditions [43]. The DN-S group started at a lower level of pain intensity than the others; this is why even though the mean difference at follow-up is greater than in the STT group, the percentage of pain reduction is the lowest of the three groups. The group of DN-S had more pain after the first session, although 15 days later the OMT and DN-S showed no differences. This could be because the local twist response is necessary to obtain positive effects producing pain and elicit referred pain. For that reason, patients in the DN-S group could have had more pain at the beginning of the treatment [44, 45].

Ay et al. did not find differences when comparing two groups (2 mL lidocaine 1% versus dry needling) for cervical spine pain on pain intensity at 4 and 12 weeks after the treatment [21]. Other studies showed similar results on pain intensity when they compared the effect 1 week, 4 weeks, and 12 weeks after one session of three different injections (dry needling versus lidocaine 0.5% versus botulinum toxin 10–20 U) in patients with myofascial pain syndrome and headache [46]. Previous studies treating patients with MTrPs in the neck region have reported pain relief after several sessions (between 5 and 6 sessions) of ischemic compression [47–49]. These outcomes agree with our study because we obtained similar differences (15.9 mm) in the STT group. However in a study in which ischemic compression was applied after a single session of lidocaine injection over MTrPs in patients with neck pain, the intensity improved to 42 mm one week after treatment [50], but that could be because drug injection was carried out.

### 4.2. Outcomes Related to Catastrophizing

In current study we have found that patients who received OMT have more improvement of catastrophizing than DN-S and soft tissue therapy. These are valuable findings because a previous study has reported that catastrophizing and disability are an important predictor of poor outcome in patients with neck pain treated by physical therapy [51]. In a prospective cohort study, it was found that more complaints and catastrophizing showed an interaction with the success of the treatment, decreasing the OR for every point increase on the catastrophizing scale. Patients with higher catastrophizing levels were more likely to achieve treatment success with manual therapy but not with physiotherapy [52]. We obtained markedly reduced PCS values in the OMT group. However, this result should be treated with caution because we obtained a mean difference of 4.8, which is not clinically significant, as it should be at least a change of 9.1 points [53].

### 4.3. Disability

The neck disability improved significantly in the DN-S and OMT groups but not in the STT group. It has been suggested that the minimum clinically important difference required for NDI is 7 points [32, 54], although others have found higher values (10.2) for their patient population [55]. In our study, the DN-S group showed difference of 5.9 points, but the OMT group showed a higher score at 8.5 points. Nevertheless, soft tissue manual therapy group did not show statistical changes in disability. However, our results are in contrast to a recent study [50] which has reported that when ischemic compression is applied, but after lidocaine injection, it produces a statically significant decrease of disability after one week when compared with lidocaine injection only after a single intervention; the range of change was clinically relevant reaching 9.5 points of difference after treatment. According to the results obtained for the self-reported variables and pain intensity, the articular-neural approach would have a greater influence over the disability and catastrophizing of the patient than the other approaches.

### 4.4. Pressure Pain Threshold

Mechanical hypoalgesia was found over the C5-C6 level and trapezius muscle in the OMT group at post 1, post 2, and 1 week later, but not for the other two groups. The PPT results showed the minimal detectable change [56] which is defined between 0.45 Kg/cm^2^ and 1.13 kg/cm^2^, with 1.45 Kg/cm^2^ for C5-C6 and 1.63 Kg/cm^2^ for trapezius muscle. The OMT group increase was 76.7% for C5-C6 level and 63.9% for the trapezius muscle. These findings are higher than those noted in previous studies which were found to be between 22.55% [18] and 40%–56% [57]. These differences could be because in our study we designed a protocol with different techniques in manual therapy, and the others have only carried out one single technique. This indicates that hyperalgesia is decreased with the articular-neural approach (OMT) and no significant difference was observed with the other therapies. A specific and specialized physical therapy must be applied to obtain the best results in this kind of patient.

This indicates that cervical pain is decreased with the articular-neural approach and no significant difference is observed with the other therapies. Although in other studies, where the patients were instructed in self-treatment using ischemic compression for 5 days, they reported a change of 1.2 kg/cm^2^, that value is considered clinically significant but is in contrast to our study for group 2. That difference could be explained because it related to different doses of treatment. In another study [49], where ischemic compression was applied, an increase of PPT of 0.79 kg/cm^2^ was found, similar to our outcomes in the STT group. Nevertheless our results are in contrast to those of Kim et al. [50] who reported a statically significant increase of PPT after lidocaine injection (3 kg/cm^2^) and ischemic compression versus lidocaine injection only (0.67 kg/cm^2^) one week after [50].

In a recent study which compared one session of dry needling versus 10 sessions of physical therapy (superficial heat, TENS 25 min, ultrasound 5 min, and trapezius stretching), there were no statistically significant differences between the groups either one week or one month after in the pressure pain threshold [58]. Nevertheless, the pressure pain threshold of myofascial trigger point hyperalgesia improved in the OMT group. Our outcomes agree with previous studies of the group, where the patients improved on the PPT after eight sessions of IC [59]. However another study showed that PPTs in a group treated with IC were significantly greater than in an ultrasound and control group [48].

The exact mechanism by which manual therapy and dry needling modulate nociceptive information remains unknown. However, many algogenic substances and neuropeptides have been found where MTrPs occur, producing peripheral sensitization [60, 61], and neuroplastic changes in the dorsal horn soon appear when a MTrPS is developed [62]. A few studies have also found enhanced activity of brain areas of pain process when MTrPs are stimulated [63], suggesting that a central and peripheral sensitization is induced. The likely mechanism of OTM for pain relief is mean central nociceptive process. On the one hand, studies have demonstrated that manual therapy could activate inhibitory descending pain system and produce hypoalgesic effects, suggesting a mechanism mediated by the periaqueductal gray via serotoninergic and noradrenergic activation [64–67]. On the other hand, decreased temporal summation following NM is applied suggesting that the dorsal horn of the spinal cord could be involved in hypoalgesic effects in healthy patients [68, 69]. It has been suggested that facet and myofascial trigger point nociceptors could be connected in the spinal cord. Therefore if facet joint pain is eliminated, then the pain of myofascial trigger point can be relieved [16, 70, 71] but not vice versa (to our knowledge there has not been any study where the cervical facet joint pain can be controlled with a trigger point injection).

### 4.5. Cervical Range of Motion

There were statistically significant differences between groups in CROM for flexion and extension the OMT group being the one that increased more than the others at posttreatments (mean difference of 13° for flexion and 8.8° for extension). The range gained exceeded the minimal detectable change, which is established at 6.5° for flexion and 5.1° for extension [35].

This outcome agrees with other studies which compared efficacy of local anesthetic injection versus dry needling on the trapezius muscle; all groups improved at CROM in patients with myofascial pain syndrome at 4 weeks and 12 weeks after a single intervention [21].

A previous study where cervical facet joint injection was used in patients with cervical myofascial pain showed that patients who received facet injection improved in CROM and experienced reduced pain intensity during 1 year of follow-up. Other previous studies have found similar effects on myofascial pain syndrome when injection into muscles of the cervical region was carried out [71, 72]. Another study found that patients with myofascial trigger points in the cervical region improved after high velocity and low amplitude were applied to cervical spine [16].

In relation to the STT group, few studies have demonstrated an increase in ROM after a single or several sessions of ischemic compression [49, 73, 74]. Cagnie et al. [59] reported that the patients treated with IC resulted in a significant improvement in flexion, extension, and side-bending immediately after eight sessions.

However, the results of this study support the results of the OMT group because joint mobilization techniques improve the pain in patients with myofascial pain syndrome [70].

In observational studies it has been reported that active MTrPs are more common in patients with cervical root compression than in healthy controls (51.2% of the patients with cervical radiculopathy). These findings suggest to us that neural pain could be involved in the MTrPs, and for this reason in our study the patients who received neural mobilization techniques can show an improvement of the outcomes. The muscle which is most frequently involved with active MTrPS was the levator scapula and the muscle more frequently involved with latent MTrPS was the upper trapezius; both muscles have been treated in the dry needling group [75].

One possible explanation of the effects of the OMT on cervical joints is that cervical facet joints are innervated by the medial branch of the posterior rami of the cervical root. When the facet joint is in disorder, the facet nociceptors transmit input to the spinal cord, and in the spinal cord other neurons can be involved; this is the underlying mechanism to explain the referred muscle pain.

### 4.6. Limitations

There are several limitations that must be taken into account in this current study. The data obtained at this research are preliminary outcomes and they are useful to calculate the sample size for future research. In order to extrapolate accurate and representative results of a future clinical trial on patients with myofascial chronic neck pain, the sample size should be of 108 patients. The necessary sample size was estimated using G^*∗*^Power 3.1.7 for Windows (G^*∗*^Power©, University of Dusseldorf, Germany) [76]. The sample size calculation was considered as a power calculation to detect between-group differences in the neck disability outcome measures. To obtain 95% statistical power (1-*β* error probability) with an *α* error level probability of 0.05, we used repeated-measured analysis of variance (ANOVA), within-between interaction, and an effect size of 0.19 to consider three groups and three measurements, generating a sample size of 30 patients per group (total simple size of 90 patients). Allowing a drop-out rate of 20% and aiming to increase the statistical power of the results, the simple size should be increased to at least 18 patients more, with a total simple size of 108 patients.

We did not include a placebo and a control group, so we are not able to know the influence of others factors like the natural evolution of the neck pain or any nonspecific effects of the treatment. However, it has been reported in the literature that manual therapy treatment is superior to a control group [14, 77] and DN is superior to a placebo, decreasing the pain in myofascial pain patients in the upper quadrant [78]. Only two sessions were applied to patients, and in clinical practice more sessions are provided; thus more sessions should be included in future studies. We only collected data for a short follow-up of 1 week; perhaps a longer follow-up time could change the course of evolution. In spite of attempts to assess the changes of catastrophizing, many other psychological variables may be assessed such as depression, anxiety, or kinesiophobia because these are involved in chronic neck pain. Only two muscles were treated in DN-S and STT groups, but myofascial trigger points of other muscles are involved with symptoms in patients with chronic neck pain. Future studies should continue to evaluate the effectiveness of various therapies in patients suffering myofascial chronic neck pain with long-term follow-up periods, including placebo and a control group. Data of the neck pain intensity should be taken with caution because the study started with a significant difference between groups at the VAS baseline outcomes.

## 5. Conclusions

The results showed that all groups experienced an improvement in neck pain intensity in the follow-up period. However, only OMT and SST had an immediate effect on reducing neck pain intensity after the first session and second session. The OMT techniques decreased mechanical hyperalgesia and pain catastrophizing and increased the cervical ROM in patients with cervical myofascial pain syndrome. In addition, the DN-S and OMT approaches showed a short term effect reducing neck disability, but only the reduction of disability in the OMT group can be considered clinically relevant.

## Figures and Tables

**Table 1:** Demographic and clinical data of three groups at the beginning of the study.

	DN-S group *N* = 12	STT group *N* = 12	OMT group *N* = 12	*P* value
Gender (male/female)	3/9	2/10	2/10	0.83
Age (years)	53.9 ± 12.7 (45.8 to 62)	45.8 ± 15.4 (36 to 55.6)	48.7 ± 10.2 (42.2 to 55.2)	0.31
Height (cm)	159.1 ± 7.4 (154.4 to 163.9)	160.8 ± 8.2 (154.4 to 163.9)	162.5 ± 6.9 (158.1 to 167)	0.52
Weight (kg)	71.1 ± 8.3 (65.8 to 76.4)	69.5 ± 17.3 (58.5 to 80.5)	60.1 ± 9.8 (53.8 to 66.4)	0.08
PCS	19.25 ± 6.48 (15.13 to 23.36)	17.50 ± 4.58 (14.58 to 20.41)	18.33 ± 4.27 (15.61 to 21.04)	0.71
NDI (points)	17.5 ± 5.5 (14 to 21.1)	17.5 ± 4.5 (14.6 to 20.3)	18.9 ± 3.4 (16.7 to 21)	0.69
Pain duration (months)	10.0 ± 2.9 (8.2 to 11.9)	11.8 ± 4.4 (9 to 14.6)	14.0 ± 3.6 (11.7 to 15.4)	0.12
Pain intensity (VAS)	33.8 ± 11.7 (26.3 to 41.3)	50.2 ± 17.6 (39 to 61.4)	42.1 ± 16.2 (31.8 to 52.5)	0.04^*∗*^

Values are expressed as mean ± standard deviation (95% confidence interval). ^*∗*^ *P* < 0.05.

**Table 2:** Comparison of changes in PSC and NDI over time for each treatment group.

Measure	Groups	Baseline mean (SD)	Follow-up mean (95% CI)	Follow-up versus baseline	
				Mean difference (95% CI)	*P* value
PSC	DN-S	19.2 ± 6.4	18.2 (15.3 to 21.1)	−1.0 (−3.6 to 1.6)	0.45
	STT	17.5 ± 4.5	16.4 (13.4 to 19.3)	−1.1 (−3.7 to 1.5)	0.41
	OMT	18.3 ± 4.2	13.1 (10.1 to 16.0)	−5.2 (−8.0 to −2.6)	0.001^*∗*^

NDI	DN-S	18.0 ± 5.4	12.2 (8.6 to 15.6)	−5.8 (−9.1 to−2.5)	0.001^*∗*^
	STT	17.4 ± 4.8	15.2 (11.6 to 18.7)	−2.2 (−5.5 to 1.0)	0.17
	OMT	18.5 ± 3.2	10.0 (6.5 to 13.5)	−8.6 (−11.8 to −5.2)	0.001^*∗*^

PSC = pain catastrophizing scale, NDI = neck disability index, DN-S = dry needling + stretching, STT = soft tissue techniques, OMT = orthopedic manual therapy, and 95% CI = 95% confidence interval. ^*∗*^ *P* < 0.001.

**Table 3:** Comparison of changes in VAS over time for each treatment group.

Measure	Groups	Baseline mean ± SD	Post 1 mean (95% CI)	Post 2 mean (95% CI)	Follow-up mean (95% CI)	Follow-up versus baseline		Post 1 versus baseline		Post 2 versus baseline	
						Mean difference (95% CI)^b^	*P* value	Mean difference (95% CI)^b^	*P* value	Mean difference (95% CI)^b^	*P* value
VAS	DN-S	33.8 ± 11.7	39.7 (28.4 to 51.0)	36.0 (24.0 to 48.0)	13.3 (3.9 to 22.7)	20.5 (7.0 to 33.9)	0.001^*∗*^	−5.9 (−5.9 to 17.7)	1.0	−2.1 (−14.1 to 18.4)	1.0
	STT	50.2 ± 17.7	38.2 (26.0 to 49.4)	30.1 (18.0 to 42.1)	34.3 (24.9 to 43.7)	15.9 (2.4 to 15.0)	0.001^*∗*^	−12.0 (−23.9 to −0.2)	0.04^*∗*^	−20.1 (−36.4 to −3.8)	0.01^*∗*^
	OMT	42.1 ± 16.3	29.8 (18.5 to 41.1)	23.0 (10.8 to 35.0)	9.4 (0.03 to 18.8)	32.7 (19.3 to 46.2)	0.001^*∗*^	−12.3 (−24.2 to −0.4)	0.03^*∗*^	−19.2 (−35.5 to −3.0)	0.01^*∗*^

VAS = visual analog scale, DN-S = dry needling + stretching, STT = soft tissue techniques, OMT = orthopedic manual therapy, and 95% CI = 95% confidence interval. ^*∗*^ *P* < 0.05.

^b^Mean diff = difference among groups for the least squares mean (adjusted for baseline and missing data).

**Table 4:** Nonparametric tests of outcome data of pressure pain thresholds.

Variable	Groups	Median (interquartile range) Pressure pain thresholds (PPT)				Friedman ANOVA	Wilcoxon (a) Base versus post 1 (b) Base versus post 2 (c) Base versus follow-up
		Baseline	Post 1	Post 2	Follow- up		
C5-C6	DN-S	2.34 (1.16–3.29)	2.42 (1.26–3.39)	2.88 (1.28–3.65)	2.09 (1.45–3.63)	0.545	(a) 0.721 (b) 0.182 (c) 0.695
	STT	2.19 (1.31–3.08)	1.74 (0.88–2.14)	1.57 (1.21–2.55)	2.25 (1.64–2.45)	0.058	(a) 0.239 (b) 0.480 (c) 0.638
	OMT	1.94 (1.44–2.19)	3.05 (2.16–4.37)	3.60 (2.59–3.74)	3.11 (2.81–3.92)	0.001	(a) 0.006 (b) 0.003 (c) 0.002
	K-W	0.612	0.008	0.019	0.011		
	Mann-Whitney *U* test						
	DN-S versus STT DN-S versus OMT STT versus OMT	0.729 0.354 0.487	0.073 0.224 0.002	0.166 0.325 0.003	0.908 0.043 0.002		

Trapezius	DN-S	3.13 (1.42–4.45)	3.20 (1.80–4.09)	3.52 (1.30–4.31)	2.85 (1.50–5.23)	0.682	(a) 0.937 (b) 0.638 (c) 0.239
	STT	2.19 (1.72–3.03)	1.84 (0.94–2.51)	1.78 (1.33–2.83)	2.49 (1.64–3.50)	0.117	(a) 0.530 (b) 0.814 (c) 0.583
	OMT	3.15 (1.62–3.24)	3.24 (2.95–4.28)	3.65 (3.21–3.68)	4.06 (3.75–4.38)	0.001	(a) 0.005 (b) 0.003 (c) 0.005
	K-W	0.453	0.008	0.072	0.012		
	Mann-Whitney *U* test						
	DN-S versus STT DN-S versus OMT STT versus OMT	0.355 0.524 0.247	0.050 0.297 0.002	0.326 0.885 0.005	0.488 0.057 0.003		

DN-S = dry needling + stretching, STT = soft tissue techniques, and OMT = orthopedic manual therapy.

K-W = Kruskal-Wallis test.

**Table 5:** Nonparametric tests of outcome data of cervical ROM (flexion and extension).

Variable	Groups	Median (interquartile range) Range of motion (ROM) in grades				Friedman ANOVA	Wilcoxon (a) Base versus post 1 (b) Base versus post 2 (c) Base versus follow-up
		Baseline	Post 1	Post 2	Follow- up		
Flexion	DN-S	38.7 (35.6–40)	40 (30.2–49.37)	41.2 (37–46.9)	45 (40–49.4)	0.085	(a) 0.448 (b) 0.079 (c) 0.009
	STT	40.0 (30.6–49.4)	46.2 (35–58.1)	50 (32.2–56.9)	40.5 (32.5–50)	0.692	(a) 0.325 (b) 0.108 (c) 0.674
	OMT	40.0 (31.5–47.5)	45.7 (35–55)	50 (50–55)	52.5 (47.5–60)	0.017	(a) 0.031 (b) 0.007 (c) 0.008
	K-W	0.804	0.626	0.070	0.036		
	Mann-Whitney *U* test						
	DN-S versus STT DN-S versus OMT STT versus OMT	0.557 0.883 0.597	0.467 0.369 0.816	0.338 0.009 0.517	0.622 0.014 0.046		

Extension	DN-S	46.25 (33.7–51)	50.7 (40–59.4)	50 (40.2–59.7)	50 (41.7–60)	0.193	(a) 0.169 (b) 0.154 (c) 0.054
	STT	42.5 (27.5–60)	41.2 (30.6–50)	45 (40–58.7)	50 (45.6–50.7)	0.633	(a) 0.580 (b) 0.635 (c) 0.422
	OMT	48.75 (40–70)	50 (40–75)	62.5 (50–65)	60 (57.5–63.7)	0.846	(a) 0.526 (b) 0.477 (c) 0.074
	K-W	0.491	0.332	0.080	0.005		
	Mann-Whitney *U* test						
	DN-S versus STT DN-S versus OMT STT versus OMT	0.794 0.257 0.381	0.210 0.816 0.195	0.839 0.044 0.062	0.726 0.022 0.001		

DN-S = dry needling + stretching, STT = soft tissue techniques, and OMT = orthopedic manual therapy.

K-W = Kruskal-Wallis test.

**Table 6:** Nonparametric tests of outcome data of cervical ROM (side-bending and rotation).

Variable	Groups	Median (interquartile range) Range of motion (ROM)				Friedman ANOVA	Wilcoxon (a) Base versus post 1 (b) Base versus post 2 (c) Base versus follow-up
		Baseline	Post 1	Post 2	Follow- up		
Side-bending	DN-S	66.2 (50–71.9)	62.2 (60–75.6)	70 (62.9–78.7)	70 (63.7–78.7)	0.005	(a) 0.858 (b) 0.028 (c) 0.014
	STT	75 (61.2–81.9)	72 (68.1–77.5)	80.5 (70–95.6)	70.5 (68.9–80.6)	0.062	(a) 0.724 (b) 0.008 (c) 0.637
	OMT	80.2 (60.1–80.5)	70 (70–72.5)	80.5 (70–86)	80 (70–95)	0.019	(a) 0.345 (b) 0.454 (c) 0.062
	K-W	0.318	0.238	0.270	0.182		
	Mann-Whitney *U* test						
	1 versus 2 1 versus 3 2 versus 3	0.154 0.257 0.543	0.098 0.268 0.597	0.104 0.324 0.602	0.663 0.092 0.154		

Rotation	DN-S	105 (88.1–116.9)	107.7 (92.5–122.5)	110.2 (89.9–129.4)	117.5 (96.2–131.9)	0.043	(a) 0.420 (b) 0.033 (c) 0.059
	STT	110 (102.5–123)	113.7 (106.2–120)	120 (110–129.6)	100 (87.5–120)	0.290	(a) 0.919 (b) 0.054 (c) 0.455
	OMT	110 (110–116.9)	120 (106.2–120)	129.5 (124–139.4)	120.7 (99.5–133.7)	0.000	(a) 0.279 (b) 0.002 (c) 0.033
	K-W						
	Mann-Whitney *U* test	0.586	0.605	0.092	0.191		
	1 versus 2 1 versus 3 2 versus 3	0.295 0.598 0.653	0.418 0.447 0.514	0.325 0.040 0.164	0.236 0.664 0.068		

DN-S = dry needling + stretching, STT = soft tissue techniques, and OMT = orthopedic manual therapy.

K-W = Kruskal-Wallis test.

## References

[1] Borghouts J. A. J., Koes B. W., Vondeling H., Bouter L. M. (1999). Cost-of-illness of neck pain in The Netherlands in 1996. *Pain*.

[2] Fernández-de-las-Peñas C., Hernández-Barrera V., Alonso-Blanco C. (2011). Prevalence of neck and low back pain in community-dwelling adults in Spain: a population-based national study. *Spine*.

[3] Simons D. G., Travell J. S. L. (1999). *Myofascial Pain and Dysfunction: The Trigger Point Manual*.

[4] Muñoz-Muñoz S., Muñoz-García M. T., Alburquerque-Sendín F., Arroyo-Morales M., Fernández-De-Las-Peñas C. (2012). Myofascial trigger points, pain, disability, and sleep quality in individuals with mechanical neck pain. *Journal of Manipulative and Physiological Therapeutics*.

[5] Fernández-de-las-Peñas C., Alonso-Blanco C., Miangolarra J. C. (2007). Myofascial trigger points in subjects presenting with mechanical neck pain: a blinded, controlled study. *Manual Therapy*.

[6] Sciotti V. M., Mittak V. L., DiMarco L. (2001). Clinical precision of myofascial trigger point location in the trapezius muscle. *Pain*.

[7] Meleger A. L., Krivickas L. S. (2007). Neck and back pain: musculoskeletal disorders. *Neurologic Clinics*.

[8] Fischer A. A. (1987). Pressure algometry over normal muscles. Standard values, validity and reproducibility of pressure threshold. *Pain*.

[9] Falla D. L., Jull G. A., Hodges P. W. (2004). Patients with neck pain demonstrate reduced electromyographic activity of the deep cervical flexor muscles during performance of the craniocervical flexion test. *Spine*.

[10] Falla D., Jull G., Hodges P. W. (2004). Feedforward activity of the cervical flexor muscles during voluntary arm movements is delayed in chronic neck pain. *Experimental Brain Research*.

[11] Zakharova-Luneva E., Jull G., Johnston V., O'Leary S. (2012). Altered trapezius muscle behavior in individuals with neck pain and clinical signs of scapular dysfunction. *Journal of Manipulative and Physiological Therapeutics*.

[12] Cooper G., Bailey B., Bogduk N. (2007). Cervical zygapophysial joint pain maps. *Pain Medicine*.

[13] Vos C. J., Verhagen A. P., Passchier J., Koes B. W. (2007). Management of acute neck pain in general practice: a prospective study. *British Journal of General Practice*.

[14] Gross A., Miller J., D'Sylva J. (2010). Manipulation or mobilisation for neck pain: a cochrane review. *Manual Therapy*.

[15] Cassidy J. D., Lopes A. A., Yong-Hing K. (1992). The immediate effect of manipulation versus mobilization on pain and range of motion in the cervical spine: a randomized controlled trial. *Journal of Manipulative and Physiological Therapeutics*.

[16] Martínez-Segura R., Fernández-de-las-Peñas C., Ruiz-Sáez M., López-Jiménez C., Rodríguez-Blanco C. (2006). Immediate effects on neck pain and active range of motion after a single cervical high-velocity low-amplitude manipulation in subjects presenting with mechanical neck pain: a randomized controlled trial. *Journal of Manipulative and Physiological Therapeutics*.

[17] Schmid A., Brunner F., Wright A., Bachmann L. M. (2008). Paradigm shift in manual therapy? Evidence for a central nervous system component in the response to passive cervical joint mobilisation. *Manual Therapy*.

[18] Sterling M., Jull G., Wright A. (2001). Cervical mobilisation: concurrent effects on pain, sympathetic nervous system activity and motor activity. *Manual Therapy*.

[19] Montañez-Aguilera F. J., Valtueña-Gimeno N., Pecos-Martín D., Arnau-Masanet R., Barrios-Pitarque C., Bosch-Morell F. (2010). Changes in a patient with neck pain after application of ischemic compression as a trigger point therapy. *Journal of Back and Musculoskeletal Rehabilitation*.

[20] Aguilera F. J. M., Martín D. P., Masanet R. A., Botella A. C., Soler L. B., Morell F. B. (2009). Immediate effect of ultrasound and ischemic compression techniques for the treatment of trapezius latent myofascial trigger points in healthy subjects: a randomized controlle. *Journal of Manipulative and Physiological Therapeutics*.

[21] Ay S., Evcik D., Tur B. S. (2010). Comparison of injection methods in myofascial pain syndrome: a randomized controlled trial. *Clinical Rheumatology*.

[22] Kamanli A., Kaya A., Ardicoglu O., Ozgocmen S., Zengin F. O., Bayik Y. (2005). Comparison of lidocaine injection, botulinum toxin injection, and dry needling to trigger points in myofascial pain syndrome. *Rheumatology International*.

[23] Peloso P., Gross A., Haines T., Trinh K., Goldsmith C. H., Burnie S. (2007). Medicinal and injection therapies for mechanical neck disorders. *Cochrane Database of Systematic Reviews*.

[24] Venâncio Rde A., Alencar F. G. PJ., Zamperini C. (2008). Different substances and dry-needling injections in patients with myofascial pain and headaches. *Cranio*.

[25] Cummings T. M., White A. R. (2001). Needling therapies in the management of myofascial trigger point pain: a systematic review. *Archives of Physical Medicine and Rehabilitation*.

[26] Jull G., Moore A. (2010). Systematic reviews assessing multimodal treatments. *Manual Therapy*.

[27] Andrade Ortega J. A., Delgado Martínez A. D., Almécija Ruiz R. (2010). Validation of the Spanish version of the neck disability index. *Spine*.

[28] Vernon H., Mior S. (1991). The neck disability index: a study of reliability and validity. *Journal of Manipulative and Physiological Therapeutics*.

[29] Simons D., Travell J., Simons L. (1999). *Myofascial Pain and Dysfunction: The Trigger Point Manual*.

[30] Sullivan M. J. L., Bishop S. R., Pivik J. (1995). The pain catastrophizing scale: development and validation. *Psychological Assessment*.

[31] García Campayo J., Rodero B., Alda M., Sobradiel N., Montero J., Moreno S. (2008). Validation of the Spanish version of the Pain Catastrophizing Scale in fibromyalgia. *Medicina Clinica*.

[32] MacDermid J. C., Walton D. M., Avery S. (2009). Measurement properties of the neck disability index: a systematic review. *Journal of Orthopaedic & Sports Physical Therapy*.

[33] Bijur P. E., Silver W., Gallagher E. J. (2001). Reliability of the visual analog scale for measurement of acute pain. *Academic Emergency Medicine*.

[34] Svensson P., Arendt-Nielsen L., Nielsen H., Larsen J. K. (1995). Effect of chronic and experimental jaw muscle pain on pain-pressure thresholds and stimulus-response curves. *Journal of Orofacial Pain*.

[35] Audette I., Dumas J.-P., Côté J. N., De Serres S. J. (2010). Validity and between-day reliability of the cervical range of motion (CROM) device. *Journal of Orthopaedic and Sports Physical Therapy*.

[36] Simons D. G. (2004). Review of enigmatic MTrPs as a common cause of enigmatic musculoskeletal pain and dysfunction. *Journal of Electromyography and Kinesiology*.

[37] La Touche R., París-Alemany A., Mannheimer J. S. (2013). Does mobilization of the upper cervical spine affect pain sensitivity and autonomic nervous system function in patients with cervico-craniofacial pain?: a randomized-controlled trial. *The Clinical Journal of Pain*.

[38] Coppieters M. W., Stappaerts K. H., Wouters L. L., Janssens K. (2003). The immediate effects of a cervical lateral glide treatment technique in patients with neurogenic cervicobrachial pain. *Journal of Orthopaedic and Sports Physical Therapy*.

[39] Vicenzino B., Neal R., Collins D., Wright A. (1999). The displacement, velocity and frequency profile of the frontal plane motion produced by the cervical lateral glide treatment technique. *Clinical Biomechanics*.

[40] Cowell I. M., Phillips D. R. (2002). Effectiveness of manipulative physiotherapy for the treatment of a neurogenic cervicobrachial pain syndrome: a single case study—experimental design. *Manual Therapy*.

[41] Cohen J. (1973). Eta-squared and partial eta-squared in fixed factor anova designs. *Educational and Psychological Measurement*.

[42] Emshoff R., Bertram S., Emshoff I. (2011). Clinically important difference thresholds of the visual analog scale: a conceptual model for identifying meaningful intraindividual changes for pain intensity. *Pain*.

[43] Ostelo R. W. J. G., Deyo R. A., Stratford P. (2008). Interpreting change scores for pain and functional status in low back pain: towards international consensus regarding minimal important change. *Spine*.

[44] Hong C.-Z., Kuan T.-S., Chen J.-T., Chen S.-M. (1997). Referred pain elicited by palpation and by needling of myofascial trigger points: a comparison. *Archives of Physical Medicine and Rehabilitation*.

[45] Hong C.-Z. (1994). Lidocaine injection versus dry needling to myofascial trigger point. The importance of the local twitch response. *American Journal of Physical Medicine & Rehabilitation*.

[46] De Venancio R. A., Alencar F. G. P., Zamperini C. (2009). Botulinum toxin, lidocaine, and dry-needling injections in patients with myofascial pain and headaches. *Cranio*.

[47] Hanten W. P., Olson S. L., Butts N. L., Nowicki A. L. (2000). Effectiveness of a home program of ischemic pressure followed by sustained stretch for treatment of myofascial trigger points. *Physical Therapy*.

[48] Sarrafzadeh J., Ahmadi A., Yassin M. (2012). The effects of pressure release, phonophoresis of hydrocortisone, and ultrasound on upper trapezius latent myofascial trigger point. *Archives of Physical Medicine and Rehabilitation*.

[49] Hou C.-R., Tsai L.-C., Cheng K.-F., Chung K.-C., Hong C.-Z. (2002). Immediate effects of various physical therapeutic modalities on cervical myofascial pain and trigger-point sensitivity. *Archives of Physical Medicine and Rehabilitation*.

[50] Kim S. A., Oh K. Y., Choi W. H., Kim I. K. (2013). Ischemic compression after trigger point injection affect the treatment of myofascial trigger points. *Annals of Rehabilitation Medicine*.

[51] Hill J. C., Lewis M., Sim J., Hay E. M., Dziedzic K. (2007). Predictors of poor outcome in patients with neck pain treated by physical therapy. *Clinical Journal of Pain*.

[52] Verhagen A. P., Karels C. H., Schellingerhout J. M., Willemsen S. P., Koes B. W., Bierma-Zeinstra S. M. A. (2010). Pain severity and catastrophising modify treatment success in neck pain patients in primary care. *Manual Therapy*.

[53] George S. Z., Valencia C., Beneciuk J. M. (2010). A psychometric investigation of fear-avoidance model measures in patients with chronic low back pain. *Journal of Orthopaedic and Sports Physical Therapy*.

[54] Young B. A., Walker M. J., Strunce J. B., Boyles R. E., Whitman J. M., Childs J. D. (2009). Responsiveness of the neck disability index in patients with mechanical neck disorders. *Spine Journal*.

[55] Cleland J. A., Childs J. D., Whitman J. M. (2008). Psychometric properties of the neck disability index and numeric pain rating scale in patients with mechanical neck pain. *Archives of Physical Medicine and Rehabilitation*.

[56] Walton D. M., Macdermid J. C., Nielson W., Teasell R., Chiasson M., Brown L. (2011). Reliability, standard error, and minimum detectable change of clinical pressure pain threshold testing in people with and without acute neck pain. *Journal of Orthopaedic & Sports Physical Therapy*.

[57] Vernon H. T., Aker P., Burns S., Viljakaanen S., Short L. (1990). Pressure pain threshold evaluation of the effect of spinal manipulation in the treatment of chronic neck pain: a pilot study. *Journal of Manipulative and Physiological Therapeutics*.

[58] Rayegani S. M., Bayat M., Bahrami M. H., Raeissadat S. A., Kargozar E. (2014). Comparison of dry needling and physiotherapy in treatment of myofascial pain syndrome. *Clinical Rheumatology*.

[59] Cagnie B., Dewitte V., Coppieters I., Van Oosterwijck J., Cools A., Danneels L. (2013). Effect of ischemic compression on trigger points in the neck and shoulder muscles in office workers: a cohort study. *Journal of Manipulative and Physiological Therapeutics*.

[60] Shah J. P., Phillips T. M., Danoff J. V., Gerber L. H. (2005). An in vivo microanalytical technique for measuring the local biochemical milieu of human skeletal muscle. *Journal of Applied Physiology*.

[61] Shah J. P., Danoff J. V., Desai M. J. (2008). Biochemicals associated with pain and inflammation are elevated in sites near to and remote from active myofascial trigger points. *Archives of Physical Medicine and Rehabilitation*.

[62] Kuan T.-S., Hong C.-Z., Chen J.-T., Chen S. M., Chien C. H. (2007). The spinal cord connections of the myofascial trigger spots. *European Journal of Pain*.

[63] Niddam D. M., Chan R.-C., Lee S.-H., Yeh T.-C., Hsieh J.-C. (2008). Central representation of hyperalgesia from myofascial trigger point. *NeuroImage*.

[64] Skyba D. A., Radhakrishnan R., Rohlwing J. J., Wright A., Sluka K. A. (2003). Joint manipulation reduces hyperalgesia by activation of monoamine receptors but not opioid or GABA receptors in the spinal cord. *Pain*.

[65] Paungmali A., O'Leary S., Souvlis T., Vicenzino B. (2004). Naloxone fails to antagonize initial hypoalgesic effect of a manual therapy treatment for lateral epicondylalgia. *Journal of Manipulative and Physiological Therapeutics*.

[66] Vicenzino B., Collins D., Benson H., Wright A. (1998). An investigation of the interrelationship between manipulative therapy-induced hypoalgesia and sympathoexcitation. *Journal of Manipulative and Physiological Therapeutics*.

[67] Paungmali A., Vicenzino B., Smith M. (2003). Hypoalgesia induced by elbow manipulation in lateral epicondylalgia does not exhibit tolerance. *Journal of Pain*.

[68] Beneciuk J. M., Bishop M. D., George S. Z. (2009). Effects of upper extremity neural mobilization on thermal pain sensitivity: a sham-controlled study in asymptomatic participants. *Journal of Orthopaedic and Sports Physical Therapy*.

[69] Bialosky J. E., Bishop M. D., Price D. D., Robinson M. E., Vincent K. R., George S. Z. (2009). A randomized sham-controlled trial of a neurodynamic technique in the treatment of carpal tunnel syndrome. *Journal of Orthopaedic and Sports Physical Therapy*.

[70] Park S.-C., Kim K.-H. (2012). Effect of adding cervical facet joint injections in a multimodal treatment program for long-standing cervical myofascial pain syndrome with referral pain patterns of cervical facet joint syndrome. *Journal of Anesthesia*.

[71] Tsai C.-T., Hsieh L.-F., Kuan T.-S., Kao M.-J., Hong C.-Z. (2009). Injection in the cervical facet joint for shoulder pain with myofascial trigger points in the upper trapezius muscle. *Orthopedics*.

[72] Kim K.-H., Choi S.-H., Kim T.-K., Shin S.-W., Kim C.-H., Kim J.-I. (2005). Cervical facet joint injections in the neck and shoulder pain. *Journal of Korean Medical Science*.

[73] Lin Y.-C., Lai C.-H., Chang W.-H., Lin J.-C., Chou S.-W. (2012). Immediate effects of ischemic compression on neck function in patients with cervicogenic cephalic syndrome. *Journal of Manipulative and Physiological Therapeutics*.

[74] Nagrale A. V., Glynn P., Joshi A., Ramteke G. (2010). The efficacy of an integrated neuromuscular inhibition technique on upper trapezius trigger points in subjects with non-specific neck pain: a randomized controlled trial. *Journal of Manual and Manipulative Therapy*.

[75] Sari H., Akarirmak U., Uludag M. (2012). Active myofascial trigger points might be more frequent in patients with cervical radiculopathy. *European Journal of Physical and Rehabilitation Medicine*.

[76] Faul F., Erdfelder E., Lang A.-G., Buchner A. (2007). G^∗^Power 3: a flexible statistical power analysis program for the social, behavioral, and biomedical sciences. *Behavior Research Methods*.

[77] Miller J., Gross A., D'Sylva J. (2010). Manual therapy and exercise for neck pain: a systematic review. *Manual Therapy*.

[78] Kietrys D. M., Palombaro K. M., Azzaretto E. (2013). Effectiveness of dry needling for upper-quarter myofascial pain: a systematic review and meta-analysis. *Journal of Orthopaedic and Sports Physical Therapy*.

